# Polymorphisms and Plasma Level of Transforming Growth Factor-Beta 1 and Risk for Preeclampsia: A Systematic Review

**DOI:** 10.1371/journal.pone.0097230

**Published:** 2014-05-13

**Authors:** Xun Li, Lin Shen, Hongzhuan Tan

**Affiliations:** Department of Epidemiology and Health Statistics, School of Public Health, Central South University, Changsha, Hunan, China; Xavier Bichat Medical School, INSERM-CNRS - Université Paris Diderot, France

## Abstract

**Background:**

Transforming growth factor-beta 1 (TGF-β1) is thought to be involved in the pathogenesis of preeclampsia (PE), but the results are inconsistent among studies. This article aims to compile an overview of the studies about the associations of TGF-β 1 polymorphism and plasma level with PE risk and to provide recommendations for future research.

**Methods and Results:**

The databases PubMed, Embase and Web of Science were searched up to December 2013. Five studies investigating the associations of four polymorphisms with the risks of PE were involved. A meta-analysis was conducted for the 869T>C polymorphism and PE risk. The results show that genotype TT of 869T>C polymorphism is a protective factor of PE (pooled odds ratio = 0.73, 95% CI: 0.56, 0.95). Eight case-control studies reported the plasma level of TGF-β 1. The substantial heterogeneity among studies may be attributed to the differences in the blood sample processing and the TGF-β 1 analysis kits. The results suggest that plasma TGF-β 1 level in the second trimester was significantly lower in the PE group than in the normal pregnancy group, but was significantly higher in the PE group during the third trimester.

**Conclusions:**

The current results support that the TGF-β 1 869 T>C polymorphism was associated with the risk of PE. However, the number of eligible studies is small and more studies are needed to clarify whether this association can be detected on larger sample sizes and different populations. Owing to the heterogeneity between studies, no conclusion on the association between plasma TGF-β 1 level and PE risk can be drawn from this review. Further studies about the TGF-β 1 levels at different stages of pregnancy and the development of TGF-β 1 assay methodology are required to reveal the role of TGF-β 1 in the pathological development of PE.

## Introduction

Preeclampsia (PE), defined as the presence of hypertension accompanied by proteinuria first appearing after 20 weeks of gestation, is a major cause of maternal and perinatal morbidity and mortality [1]. PE affects approximately 5%–8% of all pregnancies [2]. The pathophysiology of PE is not fully understood despite great efforts. The challenge to prediction, prevention and management of PE is still unsolved.

PE is characterized by abnormal vascular response to placentation that is associated with increased systematic vascular resistance, enhanced platelet aggregation, activation of the coagulation system and endothelial cell dysfunction [3]. Transforming growth factor (TGF)-β 1 is a multifunctional cytokine involved in the regulation of trophoblast invasion, proliferation, and differentiation [4], [5]. Growing evidence indicates that TGF-β 1 can be involved in the pathogenesis of PE, possibly through activation of an endothelial cell pathway [6] or regulation of systemic inflammation [7], [8]. Several genetic variants (869 T>C, 509 C>T, 800G>A and 915 G>C) related to the expression level of TGF-β 1 have been investigated for their possible association with risk of PE, but the results are inconsistent among studies[9]–[16]. The lack of reproducibility of genetic association studies is probably due to population diversity, small sample sizes in individual studies, or false-negative results with inadequate statistical power [17], [18]. The association between the plasma TGF-β 1 level and the risk of PE has been reported. Plasma TGF-β 1 level is significantly higher or lower in PE patients compared with normotensive people [19]–[30]. Many factors can affect the reported results of plasma TGF-β 1 levels, such as the assay methodology and the gestational age at sampling. Considering the insufficient evidence and inconsistent results about the association of genetic variants of TGF-β 1 and the plasma TGF levels with PE risks, a meta-analysis or systematic review is important and necessary to assess the association. The aims of this study are to overview the association studies of TGF-β 1 polymorphism and plasma levels with risk of PE, and to provide recommendations for future research.

## Methods

### Literature Search

The databases PubMed, Embase and Web of Knowledge were searched up to December 2013 for studies evaluating TGF-β 1 gene polymorphisms in PE patients. The following keywords were used: (preeclampsia OR pre-eclampsia) AND (polymorphism OR variant) AND (transforming growth factor-beta 1 OR TGF beta 1 OR TGF β1). In addition, the names of specific polymorphisms were combined with the topic “Preeclampsia”. Online databases were also searched systematically to find studies that examined the relationship between plasma TGF-β 1 level and PE risks. The following keywords were used: (preeclampsia OR pre-eclampsia) AND (transforming growth factor-beta 1 OR TGF beta 1 OR TGF β1) AND (plasma OR level OR concentration). All reference lists from the main reports and relevant reviews were hand-searched for additional eligible studies.

### Eligible Studies and Data Extraction

Studies to be included into this systematic review and meta-analysis should meet the following criteria: (1) studies investigated the relationship between PE and at least one genetic variant of TGF-β 1; or for the systematic review of TGF-beta 1 plasma concentration and risk of PE, studies included sufficient data for determining TGF-β 1 plasma level; (2) case-control studies with a case group of PE women and a control group of healthy women with uncomplicated pregnancies; (3) PE was defined as elevated blood pressure (with systolic blood pressure ≥140 mmHg and/or diastolic blood pressure ≥90 mmHg) accompanied with proteinuria measured in at least a semi-quantitative way (≥300 mg in a 24-hr urine collection and/or ≥1+ on dipstick testing), in line with International Society for Study of Hypertension in Pregnancy criteria [31]; (4) original papers contained independent data. The studies without essential information or with overlapped data were excluded.

Data were extracted independently by two reviewers in consultation with a third reviewer. For each included study, the following information was extracted: first author, year of publication, study population (country and ethnicity), and numbers of patients and controls. For genetic association studies, information was extracted from the frequency of genotypes, Hardy–Weinberg equilibrium status and genotyping methods. For studies investigating plasma TGF-β 1 level, information was extracted from method of plasma TGF-β 1 measurement, and means and standard deviation (SD) of TGF-β 1 level in each group. If standard error of mean (SEM) was reported, SD was calculated as 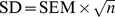.

### Statistical Methods

A meta-analysis was performed for any genetic variant that was significantly associated with PE in at least one study and has available data from at least two independent studies. Frequency of the genotypes and alleles between the PE group and the control group were compared using chi-square or Fisher exact test. The association between the polymorphism and risk of PE was estimated by calculating the pooled odds ratio (OR) and 95% confidence interval (CI), according to a general genetic model, a dominant genetic model, a recessive genetic model and an allelic model [32]–[34]. The significance of the pooled OR was determined using Z test, and *p*<0.05 was considered statistically significant.

Heterogeneity among studies was assessed using Q-test and *I^2^* statistic [35], [36]. CIs for I^2^ were also calculated [37]. For Q-test, *p*<0.1 was considered significant. With the presence of substantial heterogeneity (Q-test, *p*<0.1, and *I^2^*>50%), the random effect model was used as the pooling method, and otherwise, the fixed effect model was used. Compliance with the Hardy-Weinberg Equilibrium (HWE) among the controls in each study was checked using Chi-square test. An assessment of publication bias was planned if more than seven studies were included using funnel plot and Begg’s test; *p*<0.05 was considered as representative of statistically significant. All analyses were performed on STATA 12.0 (Stata Corporation, College Station, TX).

A meta-analysis was planned for the association studies of plasma TGF-β 1 level and PE. However, substantial heterogeneity was found among studies and therefore only a qualitative systematic review was conducted.

## Results

### Main Characteristics of All the Available Studies

**Figure 1:**
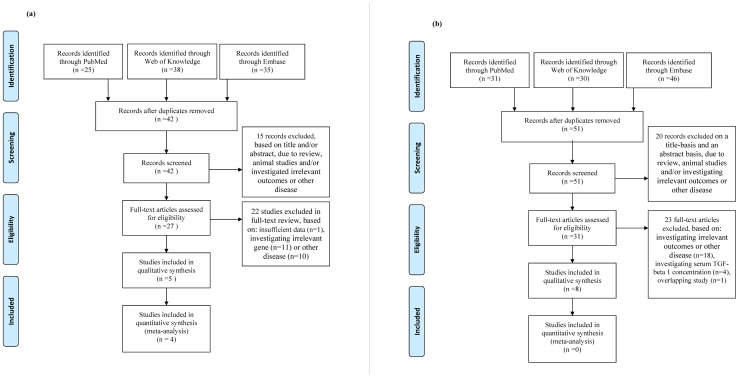
PRISMA flow diagram of study selection process (a) articles involving TGF-beta 1 genetic variants and (b) articles involving plasma concentration of TGF-beta1.

Our literature search identified five eligible studies for the association between TGF-β 1 polymorphisms and PE risk, with a total of 608 cases and 758 controls concerning four genetic variants of TGF-β 1. Kim *et al.* (2009) [9] investigated two variants (869 T>C and 915 G>C); Aguilar-Duran *et al.* (2013) [12] investigated three variants (869 T>C, 800 G>A, and 509 C>T); Stanczuk *et al.* (2007) [13] investigated two variants (869 T>C and 915 G>C); Lima *et al*. (2009) [15] investigated two variants (869 T>C and 915 G>C); and Feizollahzadeh *et al.* (2012) [10] investigated two variants (800 G>A and 509 C>T). Therefore, 869 T>C was reported in four studies, 915 G>C in three, 800 G>A in two, and 509 C>T in two. The detailed characteristics of the included studies are shown in Table 1. Among the four genetic variants, only 869 T>C was found significantly associated with PE in at least one study and provided repeated studies for estimation of pooled OR, therefore only one genetic variant was eligible for the meta-analysis. The selection of studies is shown in Figure 1 (a).

**Table 1:** Detailed characteristics of all eligible studies for the association with TGF gene polymorphisms and PE.

Gene polymorphisms	Author	Year	Country	Case	Control	Genotype*		*P* for HWE
						Case	Control	
869 T>C	Kim^a^ [9]	2009	Korean	164	182	54/79/31	80/78/24	0.47
	Aguilar-Duran^a^ [12]	2013	Mexico	175	253	31/100/44	64/120/69	0.68
	Stanczuk^b^ [13]	2007	Zimbabwe	49	86	18/18/13	34/35/17	0.16
	Lima^b^ [15]	2009	Brazilian	78	97	22/42/14	25/56/16	0.10
915 G>C	Stanczuk^b^ [13]	2007	Zimbabwe	44	76	33/11/0	66/10/0	0.54
	Lima^b^ [15]	2009	Brazilian	78	97	68/10/0	82/15/0	0.41
800 G>A	Feizollahzadeh^b^ [10]	2012	Iranian	142	140	105/30/7	98/40/2	0.35
	Aguilar-Duran^b^ [12]	2013	Mexico	175	253	152/21/2	234/18/1	0.32
509 C>T	Feizollahzadeh^b^ [10]	2012	Iranian	142	140	40/59/43	35/62/43	0.19
	Aguilar-Duran^b^ [12]	2013	Mexico	175	253	37/93/45	56/126/71	0.97

*Genotype for 869 T>C, TT/TC/CC; 915 G>C, GG/GC/CC; 800 G>A, GG/GA/AA; 509 C>T, CC/CT/TT.

a Significant differences was found in allelic frequencies between preeclamptic and control groups.

b No significant differences was found in allelic frequencies between preeclamptic and control groups.

For the association between plasma TGF-β 1 level and PE risk, eight case-control studies with a total of 627 cases and 650 controls were included (Table 2). The process of study selection is shown in Figure 1 (b).

**Table 2:** Characteristics of studies on plasma TGF-beta 1 levels and PE included in the systematic review.

Study	Year	Country	Cases	Control	Gestational stage by sampling	Test	TGF (ng/ml)^a^		*P*-value
							PE	Control	
Clausen [30]	2002	Caucasian	71	71	Second trimester	ELISA-Quantikine kit-R&D Systems	3.2 (2.0–6.1)	5.3 (3.8–7.1)	0.01
Lim [23]	2009	Korean	60	124	Second trimester	ELISA-Quantikine kit-R&D Systems	2.9±1.3	4.7±2.4	<0.001
Djurovic [25]	1997	Norway	154	76	Third trimester	ELISA-Quantikine kit-R&D Systems	5.63±1.68	4.67±1.33	0.000
Naicker [22]	2002	African	42	30	Third trimester	MEDGENIX- ELISA- BiosourceEurope SA	4.9±0.9	1.9±0.54	<0.0001
Madazli [28]	2003	Turkey	35	33	Third trimester	EIA-DRG Instruments GmbH, Germany	0.041±0.005	0.018±0.001	<0.001
Muy-Rivera [21]	2004	Peru	100	100	Third trimester	ELISA-Quantikine kit-R&D Systems	18.4±1.0	13.0±0.8	<0.001
Peracoli [24]	2008	Brazil	33	36	Third trimester	ELISA-Quantikine kit-R&D Systems	10.41±2.07	7.01±3.29	<0.05
Enquobahrie [27]	2005	Africa	132	180	After delivery	ELISA-Quantikine kit-R&D Systems	23.2±1.3	20.3±1.0	0.06

a Data are presented as mean±standard deviation or median (25–75 percentile).

### Association between 869 T>C Polymorphisms and PE Risk

**Figure 2:**
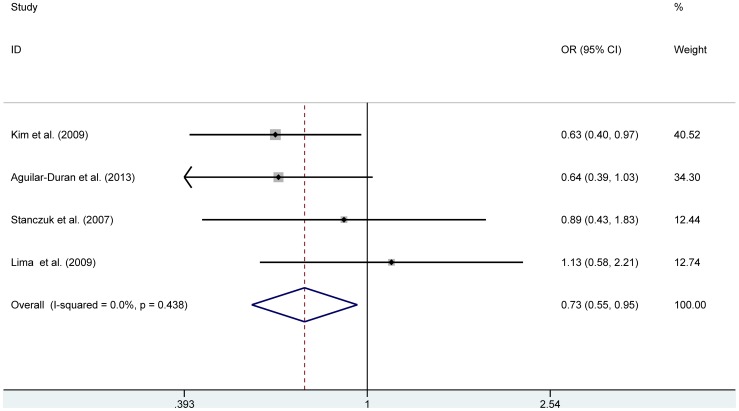
Meta-analysis for the association of 869 T>C polymorphism with PE risk under a dominant model (TT vs. (TC+CC)) using a fixed-effects model.

The meta-analysis for 869 T>C included four studies with a total of 466 cases and 618 controls. Significant associations were observed in genetic models for TT vs. TC (OR = 0.74, 95% CI [0.56, 0.99]), TT vs. CC (OR = 0.70, 95% CI [0.49, 0.99]), in the dominant genetic model (TT vs. (TC+CC)) (OR = 0.73, 95% CI [0.56, 0.95]), under the fixed effects model (Table 3, Figure 2). Publication bias may not be detected owing to the small number of available studies. A sensitivity analysis was performed by omitting one study at a time and then calculating the pooled OR of the remaining studies. After the study of Kim *et al*.(2009) [9] or Aguilar-Duran *et al.* (2013) [12] was omitted, the association between 869 T>C and PE became insignificant, suggesting that those two studies largely affected the combined results (results not shown).

**Table 3:** Main results of the pooled ORs in meta-analysis for the association between the 869 T>C polymorphism and PE.

Genetic model	Sample size		Test of heterogeneity			Test of association	
	Cases	Controls	Q	*P* (Q)	*I* ^2^ (%) (95% CI)	OR* (95% CI)	*P* (Z)
TT vs. TC	364	492	3.39	0.335	11.5 (0–86)	**0.740 (0.556–0.985)**	**0.039**
TC vs. CC	341	415	2.73	0.435	0.0 (0–85)	0.993 (0.725–1.361)	0.966
TT vs. CC	227	329	1.49	0.685	0.0 (0–85)	**0.695 (0.488–0.991)**	0.045
TT vs. (TC+CC)	466	618	2.71	0.438	0.0 (0–85)	**0.726 (0.555–0.951)**	0.020
CC vs. (TT+TC)	466	618	2.52	0.471	0.0 (0–85)	1.134 (0.843–1.526)	0.406
T vs. C	466	618	0.73	0.866	0.0 (0–85)	0.849 (0.642–1.124)	0.253

*Pooled with fixed effect model.

### Association between TGF-β 1 Level and PE Risk

Plasma TGF-β 1 levels and risk of PE were reported in eight studies, of which two studies were sampled in the second-trimester, five in the third-trimester, and one was after delivery (Table 2).

The two studies measuring TGF-β 1 levels during the second trimester showed that plasma TGF-β 1 levels were significantly lower in the PE group (3.2 and 2.9 ng/mL) than in the normal pregnancy group (5.3 and 4.7 ng/mL) (*p*<0.05 for each study). One of them (Clausen *et al.* 2002) estimated the PE risk by calculating OR and showed that during the second trimester, women with the highest levels experienced a decreased risk of PE compared to those with the lowest quartile of level (OR = 0.2, 95% CI [0.03, 0.7]).

The five studies measuring the TGF-β 1 levels during the third trimester all showed that plasma TGF-β 1 levels were significantly higher in the PE group than in the normal group. Of them, two studies reported PE risk according to upper quartile versus lower quartile of third-trimester plasma TGF-β 1 (OR = 7.2, 95% CI [2.2, 23.8] and OR = 2.5, 95% CI [1.2, 5.6]);

In the study reporting the after-delivery plasma TGF-β 1 level [21], maternal plasma TGF-beta 1 concentrations were higher in cases than in controls, falls just short of standard levels of statistical significance (23.2±1.3 versus 20.3±1.0 ng/ml, *p* = 0.06; OR = 2.5, 95% CI [1.2, 5.6], upper quartile versus lower quartile).

## Discussion

### Main Findings

Our meta-analysis shows that TGF-β 1 869 T>C was associated with PE and TT genotype of 869T>C polymorphism was a protective factor of PE. The association between other three TGE-β 1 variants (915 G>C, 800G>A and 509 C>T) and PE risk was not significant in anystudy. Plasma TGF-β 1 level in second trimester was found significantly lower in the PE group, but this association was altered in the third trimester.

### Interpretation

TGF-β family is a member of the TGF superfamily which is a collection of structurally-related multifunctional cytokines. TGF-β has three isoforms: TGF-β 1, 2, and 3. Since the first description in 1983 [38], TGF-β 1 has been suggested to be associated with a wide range of pathophysiological processes, including apoptosis of vascular endothelial cells, immune adaptation, as well as embryonic growth and development[39]–[41]. PE is pathophysiologically characterized by endothelial cell damage and dysfunction [42]. A hypothesis indicates that one or more factors released from the placenta may lead to endothelial activation and a multisystematic disorder [25], [43]. In searching for those triggers, a possible mechanistic pathway of endothelial cell activation by TGF-β 1 has been described [6]. Another hypothesis proposed that endothelial dysfunction is a part of the intravascular inflammatory reaction and PE is actually an excessive maternal inflammatory response to pregnancy [44]. The balance between regulatory T cells (Tregs) and Th17 cells is thought to be important in the development of maternal systemic inflammation in PE patients [7], [8], [45]. Tregs can induce tolerance [46], [47] and Th17 cells induce inflammation or rejection [48]. The prevalence of peripheral Tregs is decreased following PE [49]–[51] but that of Th17 cells is increased [7], [52] compared to healthy pregnant women. Since TGF can induce the differentiation of Tregs [53] and inhibit that of Th17 cells [54], the increased Th17/Treg ratio in PE patients is possibly mediated by down regulation TGF-β signaling [7]. Thus, TGF-β becomes a candidate factor that is potentially relevant to the origin of PE.

The coding region of TGF-β 1 gene is located on chromosome 19q13, and contains seven exons and six introns [4], [9]. Several polymorphisms in the TGF-β 1 gene have been reported. 869 T>C, 509 C>T and 915 G>C are significantly associated with higher, and 800G>A with lower, circulation levels of TGF-β 1 [55]–[57]. TGF 915 G>C polymorphism is related to left bentricular hypertrophy in hypertensives [58]. 896 T>C is associated with essential hypertension in Chinese [59]. Those observations indicate that polymorphisms in the TGF-β 1 gene may be involved in the pathological development of hypertension. Our meta-analysis suggests that the 869 T>C polymorphism is associated with risk of PE. However, the number of included studies is small. The results of sensitivity analysis showed that this significant association was largely affected by two studies (Kim *et al.* and Aguilar-Duran *et al.*), and therefore, the results of this meta-analysis should be interpreted with caution. Considering the small sample sizes and the relatively small numbers of studies on 509 C>T, 915 G>C and 800 G>A and risk of PE, the insignificant results do not absolutely rule out the presence of real associations with those single nucleotide polymorphisms (SNPs) and PE.

The identification of PE susceptible variants can provide new insight into its etiology. Moreover, it is an important step to individualize treatment and prevention programs according to the genetic profiles and/or clinical manifestation. The search for susceptible genes has led to an increased number of published studies associating genetic factors with PE. However, attempts to replicate these findings yielded inconsistent results. In a meta-analysis including 192 genetic association studies, 25 replicated genetic variants were identified [60]. Another meta-analysis identified 542 genetic association studies and included 22 independent meta-analyses [61]. But both studies did not include TGF-β 1 gene in the meta-analysis. Our results showed that the missed 869 T>C gene was significantly associated with risk of PE.

The reported mean plasma TGF-β 1 level ranged from 18 pg/mL to 20.3 ng/mL in normal pregnancy women. This considerable variability may be due to the differences in procedures for obtaining and processing blood samples, in the TGF-β 1 analysis kits, as well as in population characteristics. Platelet is a rich source of TGF-β 1, and platelet degranulation could happen during plasma preparation, leading to overestimation of the TGF plasma level [62]. Two included studies [22], [24] used platelet-depleted plasma and others did not indicate whether platelet was depleted. Leukocytes also contain a large amount of TGF-β 1 and it can be speculated that during plasma preparation, leukocytes secrete TGF-β 1 into the plasma [63]. Therefore, different procedures for plasma preparation between studies are a potential source of variation in reported TGF-β 1 levels. The assay methodology could be another important source of variation. Difficulties of TGF-β 1 measurement in complex biological fluids were discussed in detail in the review of Grainger *et al.* [62]. Different assays were designed to detect certain types of TGF-β 1 complexes which made some TGF-β complexes undetected by this assay. Grainger *et al.* (2000) compared the TGF-β 1 levels in the plasma of healthy subjects from 16 studies and the reported mean (or median) level ranged from less than 0.1 ng/ml to more than 25 ng/ml. Till now, there is no consensus on the plasma TGF-β 1 levels in normal humans, and no particular assay method was recommended for universal application. Although plasma TGF-β 1 was proposed as a biomarker for assessment of PE severity and significant associations were observed, heterogeneity between studies remains [25]. Thus, it may not be suitable to be introduced as a biomarker before a better assay methodology and sample preparation protocol could be developed.

To improve the comparability of results between studies, each study should provide details about the chosen assay methodology, the form of detected TGF-β 1 (active form or total), and the preparation procedure of plasma samples. Specific protocols designed to minimize contamination from platelets are available [64] and can be adopted in further studies to reduce the variation in detected TGF-β 1 levels between studies.

For individual studies in which the protocol and assay methodology should be identical in the PE group and the control group, the detected differences between groups should reflect the real differences to some degree. The plasma TGF-β 1 levels during the third trimester were significantly higher in the PE group than in the control group in all five studies, but the other two studies investigating the second trimester showed an altered association, indicating that the circulating level of TGF-β 1 during pregnancy may change in a special trend (first decreased and then elevated) in PE patients. If true, whether the decreased TGF-β 1 level during second trimester is responsible for the increased Th17/Tregs ratio and triggered the systemic inflammation in PE patients, and whether the elevated TGF-β 1 level during third trimester is one trigger or consequence of PE are unclear. Therefore, its role in the pathogenesis of PE remains intriguing and further research is needed to investigate the TGF-β 1 level throughout gestation, especially in first and second trimesters.

To the best of our knowledge, this is the first systematic review that evaluates the relationships of genetic variants and plasma level of TGF-β 1 with risk of PE. However, this study has some limitations. First, the number of studies included in the meta-analysis is comparably small (only four) and could not avoid publication bias. Although the genetic variants in PE have been investigated by hundreds of studies, TGF-β 1 is not a popular candidate gene since only five studies were identified after literature search. This is partly because TGF-β 1 was firstly identified as a candidate gene of PE as late as in 2007 [13] and its possible role in the pathogenesis of PE was described only recently [6]. Compared with other widely studied candidate genes (e.g. methylenetetrahydrofolate reductase gene C667T polymorphism first investigated in 1997 [65], [66] and now has more than 51 repeated studies [67]), the TGF-β 1 gene is a younger and less-studied one. Although the results of our meta-analysis suggest that TGF-β 1 869 T>C polymorphism was associated with risk of PE, this result was mainly determined by the study of Kim *et al*.(2009) and Aguilar-Duran *et al.* (2013). Therefore, further studies are needed. Second, it is unsuitable to conduct a meta-analysis because of significant heterogeneity among studies on plasma TGF-β 1 level and PE risk [68]. The substantial heterogeneity is probably due to the complexity of measuring methodology of TGF-β 1 level and this can also be an obstacle for further application of TGF-β 1 as a clinical indicator. However, studies show significant differences in TGF-β 1 plasma levels between PE patients and normal pregnant women, indicating that TGF-β 1 may play a role in the pathogenesis of PE. Nevertheless, this issue should be investigated by further studies.

## Conclusions

The results of this meta-analysis support that the TGF-β 1 869 T>C polymorphism is associated with the risk of PE. However, further research is needed to clarify whether this association can be detected in studies with larger sample sizes and different populations. It is difficult to conclude from the existing association studies on plasma TGF-β 1 level and PE risk owing to the between-study heterogeneity. Nevertheless, much evidence supports that the third trimester plasma TGF-β 1 level is significantly higher in PE patients compared with the control group. Further studies measuring the TGF-β 1 level at different stages of pregnancy are required to reveal the role of TGF-β 1 in the pathological development of PE.

## Supporting Information

Checklist S1 PRISMA Checklist
(DOC)
